# Identification of a GA-Related *Cis*-Element Regulating Male Peduncle Elongation in Papaya

**DOI:** 10.3390/plants15020209

**Published:** 2026-01-09

**Authors:** Julie Nguyen-Edquilang, Jingjing Yue, Ray Ming

**Affiliations:** 1 FAFU and UIUC-SIB Joint Center for Genomics and Biotechnology, Fujian Provincial Key Laboratory of Haixia Applied Plant Systems Biology, Fujian Agriculture and Forestry University, Fuzhou 350002, China; 2 Department of Plant Biology, University of Illinois at Urbana-Champaign, Urbana, IL 61801, USA

**Keywords:** papaya, *CpSVP*, GA response, peduncle elongation

## Abstract

Papaya (*Carica papaya* L.) is a tropical trioecious crop with males, hermaphrodites, and females. There is a sequence difference between male and hermaphrodite *SHORT VEGETATIVE PHASE* (*CpSVP*), making *SVP* a strong candidate gene controlling peduncle length in papaya. To study the spatial and temporal expression and function of *CpSVP* in *Arabidopsis*, we constructed a translation fusion structure based on the native promoter of *SVP* in papaya. In the 2kb promoter, strong GUS staining was observed in the floral organs and pedicels. In the 1kb promoter, there is no GUS expression in the floral organs, and it is barely detectable in pedicels. Removal of a GA responsive P-box *cis*-element in the 1kb promoter enhanced expression in the floral organs and pedicels, and elongated pedicels. In the transgenic *Arabidopsis* plants expressing the male *CpSVP* allele, there was an increase in pedicel length, but not in the plants expressing the hermaphrodite *CpSVP* allele. *CpSVP*-Y is capable of pedicel elongation, with no defects in reproductive organs. These findings imply that *CpSVP*-Y and this P-box play a major role in peduncle elongation but not sex determination in papaya.

## 1. Introduction

Papaya (*Carica papaya* L.) is a trioecious (males, hermaphrodites, and females) tropical plant, and its sex types are controlled by a pair of sex chromosomes that are approximately 7 million years old [1]. There are two Y chromosomes: Y determines males, and Yh for hermaphrodites. These two Y chromosomes diverged about 4000 years ago with only 0.4% sequence difference between them [2]. This relatively small sex determination region is 3.4 Mb and 8.1 Mb on the X and Y/Yh chromosome, respectively, and contains the sex determination genes as well as genes responsible for sex-linked traits such as peduncle length [1,3]. Male papayas are the only sex type that possess a long peduncle; therefore, the gene responsible for the peduncle length is located on the male Y chromosome, and not on the hermaphrodite Yhor female X chromosome [4]. The long male peduncle resulted in approximately 400 times more pollen production than hermaphrodite flowers, providing a selective advantage for establishing dioecy in papaya [5]. *SHORT VEGETATIVE PHASE* (*SVP*) is annotated on the male and hermaphrodite genome, as well as on the autosome but not found on the X chromosome [1]. There are coding sequence differences of *SVP* between males and hermaphrodites as the male allele contains both MADS and K-box domains while the hermaphroditic allele contains only the latter.

MADS transcription factors are combinatorial, allowing single transcription factors to control multiple genes with various temporal and spatial expression patterns. MADS box proteins form dimers or multimers to bind unique *cis*-elements to control floral organ formation [6]. *Arabidopsis SVP*, a MADS box transcription factor, is regulated by the circadian clock and the autonomous, thermosensory, and gibberellin pathways [7]. In *Arabidopsis*, *SVP* acts as a floral repressor by forming a dimer with *FLOWERING LOCUS C (FLC)* to repress *FLOWERING LOCUS T (FT)* in the leaves and *SUPPRESSOR OF CONSTANS 1 (SOC1)* in the shoot apical meristem [7]. *AtSVP* and *AGAMOUS-LIKE24 (AGL24)* determine floral meristem identity [8]. *SVP* is expressed in vegetative tissues and at undetectable levels in the shoot apex when transitioning to flowering [9]. However, in other species, *SVP* has many other functions besides flowering-time regulation. *SVP* controls dormancy in apples [10] and kiwifruit [11]. The ectopic expression of *Medicago truncatula SVP* [12] and tobacco *SVP* [13] elongated pedicels. In *Arabidopsis* and *Medicago*, a pedicle supports a single flower. The equivalent structure in papaya is a peduncle, which supports an inflorescence.

Gibberellic acid (GA) is a plant hormone that plays an important role in plant growth and development. It is involved in many physiological processes, including seed germination, stem elongation, flowering, and senescence [14]. GA binds to receptor GID1, which then causes binding of the GID1-GA complex to DELLAS, leading to GA degradation via the ubiquitin–proteasome pathway [15]. Application of GA to papaya flowers increased peduncle length with no changes in sex [4].

In this study, we described the spatial and temporal expression pattern of *CpSVP-Y* and *CpSVP-Y^h^* in *Arabidopsis*. Additionally, we investigated the role of GA-related *cis*-element in the promoter region in regulating both transcriptional activity and phenotypic traits. Four such elements were identified as repressors; their removal specifically upregulated GUS transcript levels in mature pedicels. Moreover, deletion of these *cis*-elements led to varying increases in pedicel length. Both *CpSVP-Y* and *CpSVP-Y^h^* are transcriptionally expressed in restoring flowering time floral organs, pedicels, and leaves under the regulation of their native 2kb promoters. However, only the *CpSVP-Y* allele is capable of inducing pedicel elongation. *CpSVP-Y* is capable of pedicel elongation, with no defects in reproductive organs when it was expressed under the native promoter. We dissected the *CpSVP-Y* promoter and found that the removal of a pyrimidine box (P-box) at −461 bp increased expression in the floral organs and elongated pedicels. These findings imply that *CpSVP-Y* and this P-box play a major role in peduncle elongation but not sex determination in papaya.

## 2. Results

### 2.1. Expression Pattern of CpSVP Driven Under Its Native Promoter

To determine the spatial and temporal expression of *CpSVP* and to determine its role in pedicel elongation, 1kb and 2kb upstream promoter sequences (from ATG start site) from male and hermaphroditic alleles of *CpSVP* were fused to *CpSVP-Y* and *CpSVP-Y^h^*, respectively, and subsequently fused to the β-glucuronidase (GUS) reporter gene. The promoter-less GUS construct (pMDC162) was used as a negative control.

**Figure 1:**
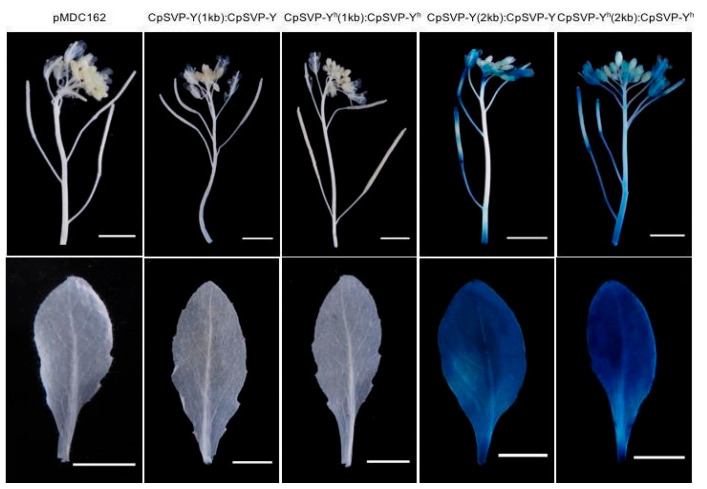
Histochemical staining of translational fusion constructs (designs in which a promoter drives the expression of a target gene coding sequence translationally fused to GUS, thereby producing a single fusion protein). Scale bars are 5 mm.

**Figure 2:**
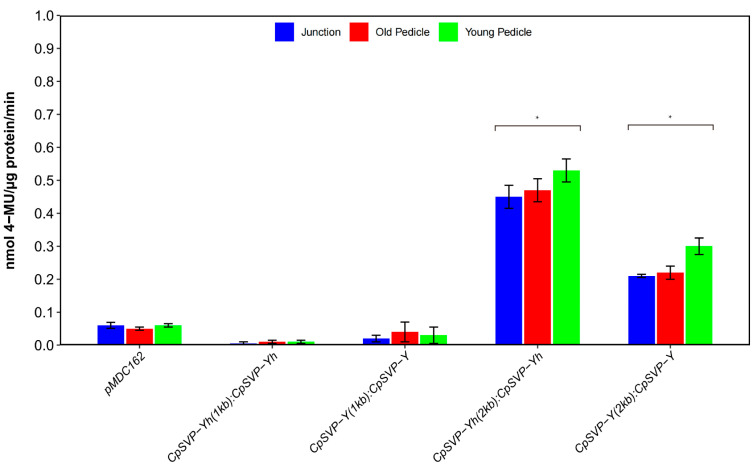
GUS activity of translational fusion constructs. Values are represented as mean ± standard error (mean ± SE). Results were compared to the pMDC162 empty vector and analyzed using Student’s *t*-test (* *p* < 0.05). Values were obtained from four biological replicates and three technical replicates.

In *CpSVP-Y (2kb): CpSVP-Y* and *CpSVP-Y^h^ (2kb): CpSVP-Y^h^* plants where GUS expression was driven by 2kb promoter sequence, GUS is highly expressed in leaves, pedicels, pollen grains, stigma, stamen, ends of siliques, and sepals (Figure 1). Old pedicels showed a stronger GUS signal than young pedicels for all *CpSVP* transgenic lines (Figure 1). For constructs driven by 1kb promoter sequence, GUS expression from *CpSVP-Y (1kb):CpSVP-Y* and *CpSVP-Y^h^ (1kb): CpSVP-Y^h^* transgenic plants was only faintly detected in the leaves and pedicels and absent from the reproductive organs (Figure 1). To quantify the GUS activity, the MUG assay was performed on three tissues: junction (where the old pedicel meets the main inflorescence stem), and two stages of pedicels (young pedicel–open flowers with no siliques, and old pedicel–with siliques present). Since *CpSVP-Y* and *CpSVP-Y^h^* promoters are highly similar with only two nucleotide differences at −159 bp, we expect no differences in the expression pattern between them. As expected, there was no difference spatially or temporally in the histochemical stain between *CpSVP-Y (1kb*) and *CpSVP-Y^h^ (1kb)*, and between *CpSVP-Y(2kb)* and *CpSVP-Y^h^ 2kb)* (Figure 1). The same pattern was observed when quantifying promoter activity fluorometrically (Figure 2). In transgenic plants driven by the 2kb promoter of *CpSVP-Y* or *CpSVP-Y^h^* plants, GUS expression was more intense in the pedicels and leaves and there was additional presence of additional signals in reproductive organs when compared to their counterparts, transgenic plants driven by 1kb promoter. This suggests there might be *cis*-element(s) located between the 1kb and 2kb promoter region that enhance the *CpSVP* expression in the leaves and pedicels, and that it is necessary for expression in the carpels and stamens.

### 2.2. Identification of CpSVP Promoter Cis-Elements Controlling Tissue-Specific Expression

To dissect the *cis*-element(s) that contributed to the *CpSVP* expression pattern difference between *CpSVP-Y (1kb): CpSVP-Y* and *CpSVP-Y (2kb): CpSVP-Y*, we scanned the 2kb promoter region using PLACE (https://www.dna.affrc.go.jp/PLACE/, accessed on 4 January 2026) [16] and PlantCARE (http://bioinformatics.psb.ugent.be/webtools/plantcare/, accessed on 4 January 2026) [17]. *Cis*-elements located between the ATG start site and 1kb region are light-responsive elements, Opaque-2 (*O2*) elements needed for zein metabolism regulation, TCA-elements involved in salicylic acid responsiveness, low-temperature responsive elements, binding sites for *MYELOBLASTOSIS (MYB)* transcription factors, defense and stress responsiveness, and a P-box involved in gibberellin acid (GA) response. *Cis*-elements located between 1kb and 2kb region are anaerobic induction elements, CAT-box for meristem expression, light-responsive elements, low-temperature responsiveness elements, binding sites for *MYB* and *WRKY* family of transcription factors, a homeodomain–leucine zipper (HD-ZIP) protein binding site, and the GA response element (GARE) and P-Box, which are both involved in GA response (Figure S1).

**Figure 3:**
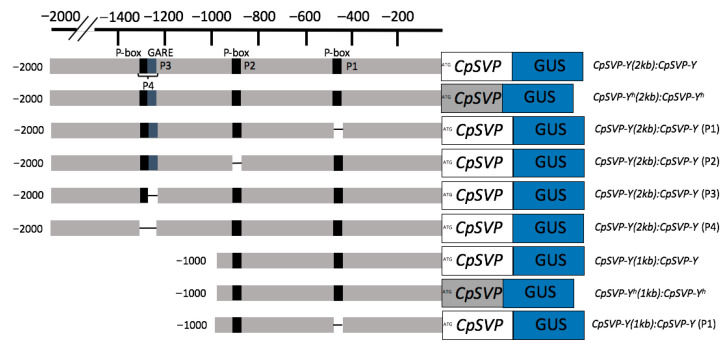
Schematic diagram of translational fusion constructs and mutagenized translational fusion constructs. Gray boxes depict the *CpSVP-Y^h^* gene and white boxes depict the *CpSVP-Y* gene. The black and blue boxes indicate predicted cis-regulatory elements, including P-box and GARE motifs.

**Figure 4:**
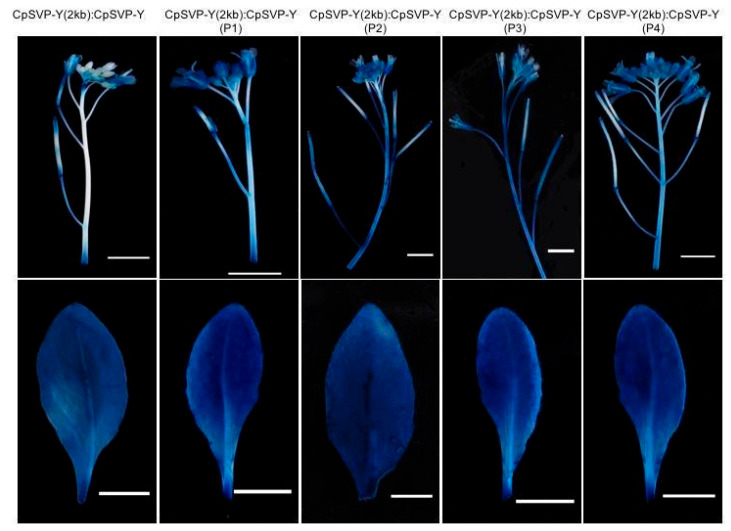
Histochemical staining of *CpSVP-Y (2kb): CpSVP-Y* translational fusion lines and mutagenized *CpSVP-Y (2kb): CpSVP-Y* lines. Scale bars are 5 mm.

**Figure 5:**
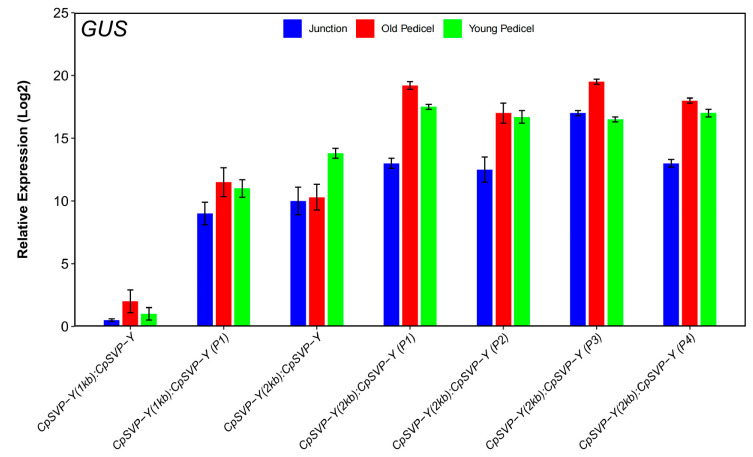
Relative expression of GUS transcript levels in translational and mutagenized lines. Values are represented as mean ± standard error (mean ± SE). The qPCR expression level of each construct was normalized to *ACTIN* and *EIF1* expression in wild-type Col-0 plants. Values were obtained from three biological replicates and two technical replicates.

As exogenous application of GA3 to papaya flowers caused pedicels to elongate [4], there might be a correlation between GA and pedicel length through the involvement of *CpSVP*. Identified in barley, a GA response complex (GARC) is usually composed of three motifs: the GA response element (GARE) TAACAAA, the pyrimidine box (P-box) CCTTTT, and the Amy box TATCCAC [18,19,20]. There were four GA *cis*-elements identified in the 2kb *CpSVP* promoter region, termed P1–P4 based on their proximity to the ORF, with P1 being closest to the ATG start codon (Figure 3). P1 and P2 correspond to P-box motifs (CCTTTT), P3 corresponds to a GA-responsive element (GARE; TAACAAA), and P4 is a composite element containing both a P-box and a GARE. In this study, we collectively refer to these motifs as the GA-related *cis*-elements P1–P4 (Figure 3). We did not identify a complete GARC, only a partial GARC in the P4 element. We used site-directed mutagenesis (SDM) to delete these four GA-related *cis*-elements P1–P4 from the *CpSVP-Y* promoter. *CpSVP-Y (2kb):CpSVP-Y* has high GUS expression in the newly emerged floral buds and open flowers, and no expression in the mature flowering buds before opening (Figure 1). When removing P1–P4 elements individually from *CpSVP-Y (2kb):CpSVP-Y*, there was an increase in GUS expression in all floral buds, main inflorescence stem, and older pedicels (Figure 4). In all *CpSVP-Y (2kb)* mutagenized lines, GUS transcript expression was increased in the old pedicels (Figure 5). This suggests that P1–P4 act as repressors in a tissue-specific manner, as removal of these elements increased GUS transcript expression in the old pedicels specifically.

**Figure 6:**
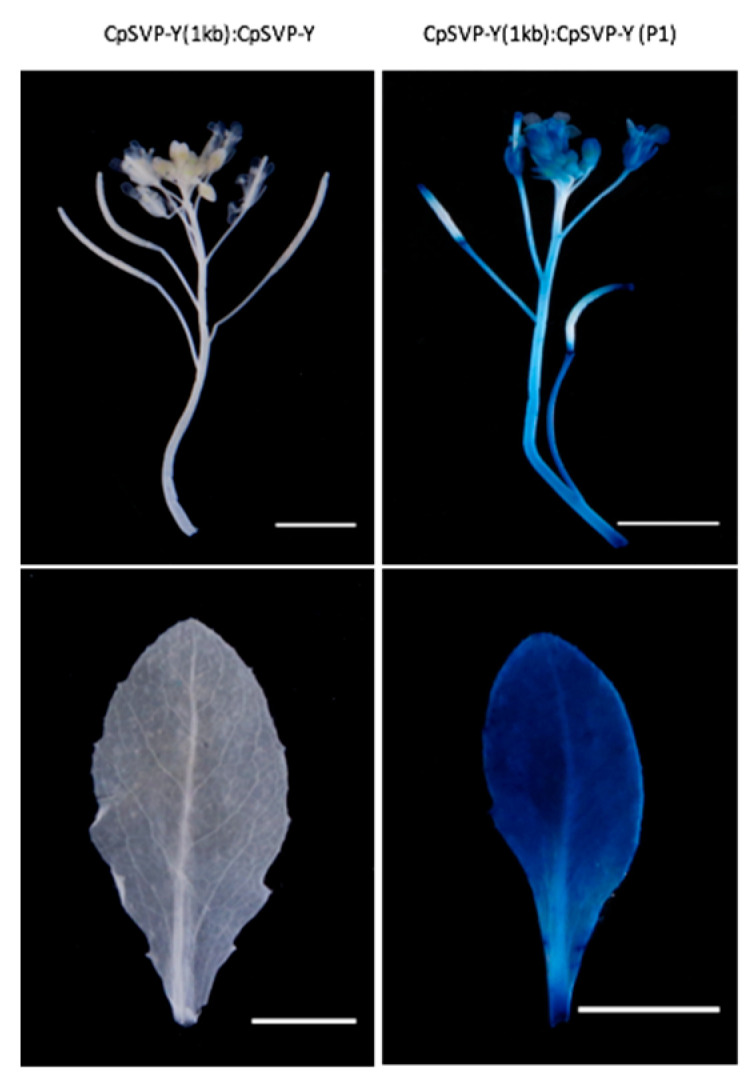
Histochemical staining of *CpSVP-Y (1kb): CpSVP-Y* and P1-removed *CpSVP-Y (1kb): CpSVP-Y* lines. Scale bars are 5 mm.

**Figure 7:**
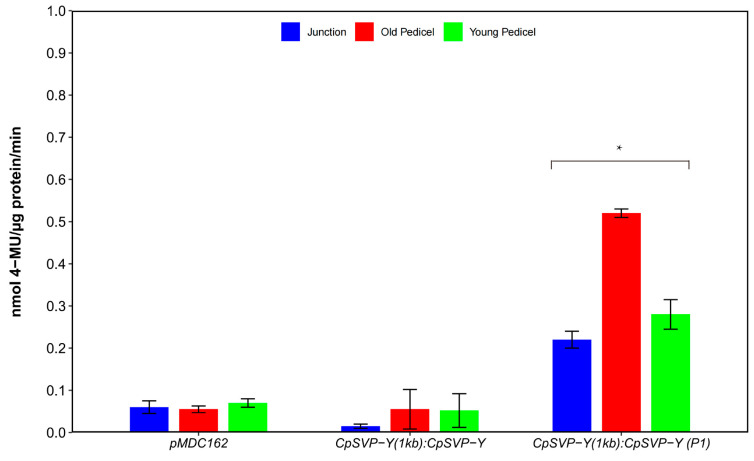
Relative expression of GUS transcript levels in translational and mutagenized lines. Values are represented as mean ± standard error (mean ± SE). The qPCR expression level of each construct was normalized to *ACTIN* and *EIF1* expression in wild-type Col-0 plants and analyzed using Student’s *t*-test (* *p* < 0.05). Values were obtained from three biological replicates and two technical replicates.

**Figure 8:**
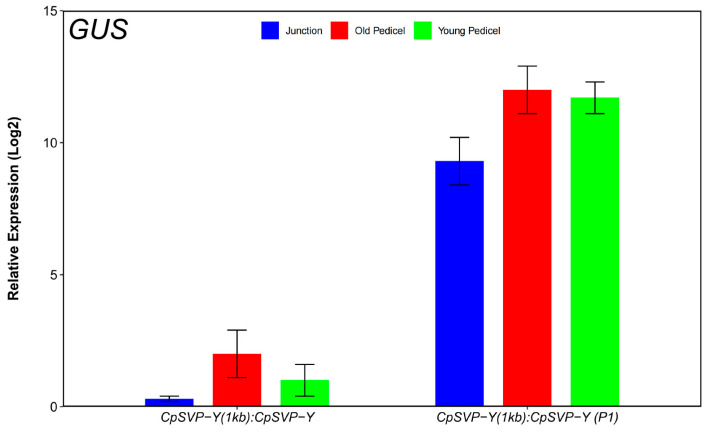
Quantitative GUS activity of *CpSVP-Y (1kb):CpSVP-Y* and the P1-deleted *CpSVP-Y (1kb):CpSVP-Y* lines. Values are represented as mean ± standard error (mean ± SE). Values were obtained from three biological replicates and two technical replicates.

Removing the P1 GA *cis*-element from *CpSVP-Y (1kb):CpSVP-Y* restored GUS activity in the reproductive organs and enhanced expression in the pedicels and leaves, with a similar phenotype as *CpSVP-Y (2kb)*: *CpSVP-Y* plants (Figure 1 and Figure 6). Quantification of GUS transcript and protein levels further confirmed the increment of GUS expression at pedicel tissues when compared to construct without P1 deletion (Figure 7 and Figure 8).

### 2.3. Cis-Element on CpSVP Promoter Regulates Pedicel Elongation

Male papaya plants are the only sex type that bear a long pedicel with large numbers of flowers situated on pedicels. Our studies showed that the *CpSVP-Y* gene can regulate pedicel growth in *Arabidopsis*, but not the *CpSVP-Y^h^* gene when it was expressed constitutively or driven by its native promoter. To determine if the *CpSVP-Y* promoter plays an additional role in pedicel elongation spatially and temporally, we introduced the intact translational fusion constructs native promoter fused with gene—C*pSVP-Y (1kb): CpSVP-Y* and C*pSVP-Y (2kb): CpSVP-Y* and the translational fusion constructs with deleted *cis*-elements [C*pSVP-Y (1kb): CpSVP-Y* (P1), C*pSVP-Y (2kb): CpSVP-Y* (P1), C*pSVP-Y (2kb): CpSVP-Y* (P2), C*pSVP-Y (2kb): CpSVP-Y* (P3), and C*pSVP-Y (2kb): CpSVP-Y* (P4)] into *Arabidopsis* Col-0 background (Figure 3). C*pSVP-Y (2kb): CpSVP-Y* had a mean pedicel length of 7.51 mm. Removing P1, P2, P3, or P4 elements resulted in an average pedicel length of 7.37 mm, 7.87 mm, 7.82 mm, and 8.66 mm, respectively (Figure S2). Deleting the P4 element increased the pedicel length by 15% when compared to the C*pSVP-Y (2kb): CpSVP-Y* transgenic plants (*p* < 0.01, Student’s *t*-test). Upon investigation of GUS transcript expression in junction, young, and old pedicels, the expression levels of the P1 to P4 deletion lines were very similar, suggesting that *CpSVP-Y* expression might also be comparable. This suggests that the significant pedicel elongation in the P4 deletion line might not be attributed to an increase in transcript expression but rather to enhancements at the post-translational and/or protein level. C*pSVP-Y (1kb): CpSVP-Y* had a mean pedicel length of 7.59 mm and C*pSVP-Y (1kb): CpSVP-Y* (P1), and the average pedicel length is 8.11 mm (Figure S3). Removal of P1 element from C*pSVP-Y (1kb):CpSVP-Y* resulted in a 7% increase in pedicel length (*p* < 0.05, Student’s *t*-test). As the 2kb promoter region contains an additional *cis*-element that could be bound by transcription factor(s) for repression, removing the P1 element from C*pSVP-Y (2kb): CpSVP-Y* did not increase pedicel length, as it did in C*pSVP-Y (1kb): CpSVP-Y* (P1) plants.

### 2.4. CpSVP-Y Delays Flowering

*SVP* regulates flowering in a dosage-dependent manner by repressing *FLOWERING LOCUS T* (*FT)* [21]. In this study, we observed the conserved role of *CpSVP* in controlling flowering time. *CpSVP-Y (1kb): CpSVP-Y* and *CpSVP-Y (2kb): CpSVP-Y* plants flowered later than Col-0 (*p* < 0.01, Student’s *t*-test, Table S3). Significantly, deletion of the P1 element from either the *CpSVP-Y (1kb): CpSVP-Y* and *CpSVP-Y (2kb): CpSVP-Y* construct abolished this late-flowering phenotype, restoring flowering time to that of Col-0. This could be due to the change in spatial expression as well as reduced or no *CpSVP* expression in the shoot apical meristem. Removal of P2, P3, and P4 elements from *CpSVP-Y (2kb): CpSVP-Y* also delayed flowering compared to Col-0, but similar to the *CpSVP-Y (2kb): CpSVP-Y* plants (Table S3). The removal of the P1 element downregulates *CpSVP* expression in the shoot apical meristem, which abolishes the late-flowering phenotype. This indicates that the P1 element is both unique and necessary for *CpSVP-Y* function in flowering time control. It likely acts as a transcription factor binding site and plays a critical role in activating *CpSVP-Y*-mediated regulation of flowering.

### 2.5. Elucidating the Mechanism for Pedicel Elongation

To understand how the removal of GA *cis*-elements affects GA signaling, we examined the transcript levels of *AtGA20ox2* in the native promoter lines (C*pSVP-Y (1kb): CpSVP-Y* and C*pSVP-Y (2kb): CpSVP-Y*) and mutated lines. *AtGA20ox2* plays a key role in GA biosynthesis, encoding the rate-limiting enzyme that catalyzes the second-to-last step of bioactive GAs [22,23]. The mutation of these GA *cis*-elements had minimal effect on *AtGA20ox2* expression (Figure S4), suggesting they do not affect GA signaling. GUS is lowly expressed in C*pSVP-Y (1kb): CpSVP-Y* and highly expressed in C*pSVP-Y (2kb): CpSVP-Y* in all tissue types, but both show similar *AtGA20ox2* expression pattern, suggesting no direct correlation between *CpSVP* and *AtGA20ox2* expression (Figures S5 and S6). In addition, *AtGA20ox2* expression is upregulated in *CpSVP-Y (2kb): CpSVP-Y* old pedicels, and removal of P1–P4 elements individually did not alter its expression.

In *Arabidopsis*, *ASYMMETRIC LEAVES 2* (*AS2*) suppresses *BREVIPEDUNCLELUS (BP)* to control pedicel length [24]. To further dissect the mechanism of peduncle elongation in papaya, we analyzed the expression patterns of these genes in transgenic *Arabidopsis* plants expressing the papaya *CpSVP-Y* gene under the control of its native promoters (1kb or 2kb), as well as in lines with mutations in the putative *cis*-elements (P1–P4). The removal of the P1 element from the *CpSVP-Y (1kb): CpSVP-Y* construct resulted in the upregulation of *AS2* in young pedicels compared to the non-mutated *CpSVP-Y (1kb): CpSVP-Y* line. Conversely, in the *CpSVP-Y (2kb): CpSVP-Y* background, the removal of P1 led to the downregulation of *AS2* in young pedicels compared to the non-mutated *CpSVP-Y (2kb): CpSVP-Y* line. This suggests that the *CpSVP* (2kb) promoter region possesses additional *cis*-regulatory elements that could be bound by transcription factor(s), which *CpSVP* (1kb) is missing. *CpSVP-Y (2kb): CpSVP-Y* and *CpSVP-Y (1kb): CpSVP-Y* transgenic plants had higher *BP* expression levels compared to the wild-type Col-0. However, mutagenesis of the GA *cis*-elements increased *CpSVP* and decreased *BP* expression in the old pedicels, resulting in elongated pedicels compared to the non-mutated lines (Figure S6), suggesting that *CpSVP* could interact with *BP* for pedicel elongation.

## 3. Discussion

### 3.1. Mutagenesis of GA-Binding Site Caused Ectopic Expression of CpSVP-Y in Floral and Vegetative Organs and Elongated Pedicels

Promoter analysis of *CpSVP-Y* identified four GA-related *cis*-elements, designated P1–P4 [P1 and P2: P-box motifs; P3: GARE motif; and P4: composite P-box + GARE]. Site-directed mutagenesis of these elements in the *CpSVP-Y (2kb): CpSVP-Y* promoter—P1 at −461 bp, P2 at −895 bp, the GARE motif at P3 (−1268 bp), and the composite P4 element (P-box + GARE) at −1272 bp—revealed that all four deletions increased GUS expression in pedicels to varying degrees (16–58%) compared to the intact *CpSVP-Y (2kb): CpSVP-Y* line in *Arabidopsis*. However, only deletion of the composite P4 element produced a notable phenotypic effect, resulting in a 15% increase in pedicel length. In contrast, removal of P1, P2, or P3 caused minimal changes in pedicel elongation, despite elevated GUS levels. Deletion of the P3 GARE motif slightly decreased GUS expression in young pedicels and at the pedicel junction (by 3% and 16%, respectively), suggesting a positive regulatory role for this motif in these tissues. Overall, the magnitude of GUS transcriptional changes does not correlate with pedicel length variation, indicating that pedicel elongation is likely influenced by additional post-transcriptional or post-translational regulatory processes.

**Figure 9:**
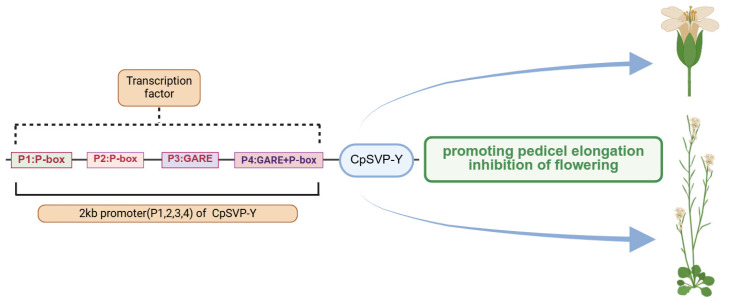
Schematic diagram of *CpSVP-Y* promoting pedicel elongation and inhibiting flowering in *Arabidopsis*. Solid lines indicate experimentally validated regulatory relationships. Dashed lines indicate putative or indirect regulatory relationships inferred from bioinformatic or correlative analyses. Arrows indicate positive regulation or activation.

Mutation of a P-box at −461 bp in *CpSVP-Y (1kb): CpSVP-Y* enhanced GUS expression in the reproductive organs and the pedicels (Figure 3, Figure 4 and Figure 5), due to the increased *CpSVP* expression indirectly assayed by GUS; pedicel length increased by 7% compared to *CpSVP-Y (1kb): CpSVP-Y*. Interestingly, removal of P1 *cis*-element from *CpSVP-Y (2kb): CpSVP-Y* did not increase pedicel length, contrasting with the increase in GUS expression, as it did for *CpSVP-Y (1kb): CpSVP-Y.* This suggests that there is an additional *cis*-regulator(s) present in the 2kb promoter and absent from the 1kb promoter, enhancing pedicel elongation. Therefore, we primarily focus on the 1kb to 2kb region of the promoter, rather than the 1kb fragment, to investigate potential regulatory elements. Removal of the P4 *cis*-element significantly increased pedicel length, pointing to a particularly strong repressive role of its cognate transcription factor in limiting pedicel growth (Figure 9). *Prolamin box binding factor* (*PBF)*, a DNA-binding with One Finger (*DOF*) transcription factor, has been identified in barley to interact with the P-box, as the complementary sequence ‘AAAG’ is the required binding site for DOF proteins [25,26]. DOF proteins are involved in many other biological processes, such as gibberellin response [27] and photoperiodic flowering [28]. The P-box at −461 bp plays a pivotal role for *CpSVP-Y.* Some *trans*-acting factor homologous to *PBF* could interact with the P-box to repress *CpSVP* expression in the reproductive organs and pedicels. DOF/PBF proteins function primarily as transcriptional regulators that bind to AAAG-containing P-box motifs, where they recruit co-regulators to modulate gene expression in GA-responsive and developmental pathways. Such DOF–P-box interactions provide a plausible mechanism through which P1 may influence *CpSVP-Y* transcription in reproductive tissues. Similarly, the maize Opaque-2 (O2) bZIP transcription factor activates target genes by binding its specific *cis*-element and often acts together with P-box and GARE motifs as part of a gibberellin response complex. Because the transcriptional output of this module depends on the spatial arrangement of these motifs, the proximity of the O2 site to the P1 P-box in the *CpSVP-Y* promoter suggests that O2-like factors may modulate GA-dependent regulation of *CpSVP* expression and pedicel elongation in *Arabidopsis*.

Both *CpSVP-Y (1kb): CpSVP-Y* and *CpSVP-Y (2kb): CpSVP-Y* had similar pedicel lengths, but significantly different GUS expression, suggesting that there are regulatory elements present on the 1kb promoter that is/are needed to elongate pedicels and enhance *CpSVP-Y* expression. This is consistent with our observation in P1-removed *CpSVP-Y (1kb): CpSVP-Y* plants, as the GUS activity was still lower than *CpSVP-Y (2kb): CpSVP-Y* plants. As *CpSVP-Y (2kb): CpSVP-Y* is still able to elongate pedicels compared to Col-0, the P4 *cis*-element will not prohibit papaya pedicel elongation completely. Earlier, we identified a TCA element associated with salicylic acid (SA) response, only found in the 1kb promoter region. In addition to the defense and abiotic stress response, salicylic acid is also involved in plant growth and development. In Iris (*Iris hollandica)*, application of SA on the iris significantly increased pedicel length [29]. In *Arabidopsis*, overexpression of *OBF-Binding Protein 3* (*OBP3)*, a SA-inducible *DOF* transcription factor, decreased growth rate in the aerial parts of the plant [30]. *OBP3* could bind to P-boxes and indirectly interact with the TCA element to alter SA levels to promote pedicel growth. Also, in the 1kb promoter region, we identified a putative binding site for *opaque-2 (O2)*, a maize endosperm-specific transcription factor that regulates storage protein zein genes. The *O2 cis*-element, P-box, and GARE form a gibberellin response complex (GARC), and the GARE motif can substitute for the *O2 cis*-element to enhance transcription in barley aleurone cells in response to GA [20]. Positioning *O2* and GARE 70 bp apart drastically reduced the basal level of GUS with or without GA induction; as the interaction of *O2 cis*-element, P-box, and GARE is positionally and spatially specific, this suggests that GARE does not function as an enhancer [20]. This *O2* element is located at −405 bp and GARE is located at −1268 bp, suggesting it is unlikely GARE will enhance transcription due to its proximity to *O2*. However, *O2* is located within distance of a P-box at −461 bp. *O2* functions as a coupling element to affect the spatial and developmental response to GA. *O2* could act as a coupling element with the P-box and GA to enhance *CpSVP* expression and elongate pedicels.

Removal of the P-box-containing GA-related *cis*-elements (e.g., P1 and P4) elongated pedicels suggesting that *CpSVP-Y* and the P-box is involved in pedicel elongation; however, there is no correlation between GUS transcripts and the 2kb-mutagenized *cis*-element lines. Pedicel length does not seem to be proportional to the transcriptional level of GUS, but potentially due to post-translational modifications and/or increased protein levels. It is worth noting that significant GUS activity was detected in the leaves, suggesting that CpSVP may play a role during vegetative development. Moreover, the coordinated enhancement of expression signals in both leaves and pedicels by the 2kb promoter indicates that the *cis*-elements located in the 1–2kb region likely function as basal transcriptional enhancers, rather than being limited to tissue-specific regulation alone. This broad expression profile underscores the potential pleiotropic functions of CpSVP in integrating growth signals across developmental stages.

### 3.2. CpSVP and the Pyrimidine Box Are Involved in Flowering

In the male and hermaphroditic (2kb) native promoter lines, *CpSVP* was highly expressed in the newly emerged floral buds, and then disappeared in the older floral buds, consistent with its role as a floral repressor in *Arabidopsis*. In addition to high *CpSVP* expression in the reproductive organs and pedicels, mutagenesis of the P-box also altered flowering time. *CpSVP-Y* plays a conserved role in controlling flowering, like its *Arabidopsis* homolog as *CpSVP-Y (1kb): CpSVP-Y* and *CpSVP-Y (2kb): CpSVP-Y* flowered later than Col-0. They differ drastically in GUS expression, suggesting that a shared regulatory element within the 1kb promoter region, which controls the spatial expression of *CpSVP*, is required for flowering-time regulation. However, mutagenesis of the P-box at P1 (−461 bp) abolished the late-flowering phenotype (Table S3), likely implying that transcriptional regulators acting via P1 dominantly suppress flowering in *Arabidopsis* and that removal of P1 releases this repression (Figure 9). Consistent with this, P1-mutated lines, which lose the localized GUS signal and show broader *CpSVP-Y* promoter activity, also exhibit reduced pedicel elongation compared with lines carrying the intact 1kb promoter, indicating that both the presence and the spatial restriction of *CpSVP-Y* expression contribute to the observed pedicel-length phenotype. Alternatively, the P1 element may be essential for the binding of transcription factor(s) that are involved in flowering-time regulation. In contrast, in the mutagenesis of the P-box at P2 (−895 bp), the GARE motif at P3 (−1268 bp), or both the P-box and GARE at P4 (−1272 bp) in *CpSVP-Y (2kb): CpSVP-Y* did not affect the late-flowering phenotype, suggesting that these three sites might not be directly involved in flowering-time regulation. Although moderate differences in GUS staining patterns were observed among the P2–P4 mutants, these changes did not translate into detectable alterations in flowering time or pedicel length, implying that the correlation between reporter activity and phenotype is not strictly linear and may involve threshold or tissue-specific effects.

*CYCLING DOF FACTOR 1 (CDF1)* transcription factor binds to the DOF binding sites, the complementary sequence of the P-box, in *CONSTANS (CO)* promoter, to repress *CO* transcription [28]. *FLAVIN-BINDING*, *KELCH REPEAT*, *F-BOX 1 (FKF1)* forms a complex with another F-box transcription factor, *GIGANTEA (GI)*, and targets *CDF1* for degradation while bound to *CO*, to activate *CO* transcription and allow *CO* to activate *FT* transcription [28,31,32]. Given that P-box (pyrimidine box) and GA-responsive GARE motifs function as *cis*-elements in multiple GA-regulated promoters, their presence in the CpSVP-Y promoter suggests that similar DOF–GA signaling modules could integrate hormonal and photoperiodic cues to fine-tune flowering and peduncle elongation in papaya. *CDF*, a *DOF* transcription factor, is capable of binding to P-boxes, as they contain the required binding sequence, to repress *CO* and *FT*, possibly coordinating the flowering transition in papaya. Removal of the P-box removes the *CDF* repressive mark on flowering, suggesting this could be how P1-removed transgenic plants lost the late-flowering phenotype. However, removal of P2–P4 *cis*-elements and *CpSVP-Y (1 and 2kb):CpSVP-Y* did not affect the late flowering phenotype, suggesting there must be additional regulatory elements present on the 1kb promoter to regulate the flowering transition. It is also possible that post-transcriptional and post-translational mechanisms contribute to the incomplete correlation between GUS expression and pedicel length. In *Arabidopsis*, SVP protein abundance and activity are modulated by environmental signals and interacting partners, adding regulatory layers beyond transcription [33]. By analogy, differences in *CpSVP-Y* mRNA stability, translation efficiency, or protein turnover, as well as regulation of CDF-like repressors, could modulate flowering time and pedicel elongation independently of the GUS reporter readout. These hypotheses may partially explain why some promoter mutations alter GUS patterns without proportionally affecting the developmental phenotype and highlight potential targets for future functional and molecular studies. The incomplete correlation observed between GUS expression levels and pedicel elongation suggests that the transcriptional changes may not be the sole determinants of this phenotype. This discrepancy could be explained by post-transcriptional or protein-level regulation, such as mRNA stability, translation efficiency, or protein modifications, which may influence pedicel elongation independently of transcriptional changes [34].

### 3.3. Potential Mechanism for Pedicel Elongation

We measured the transcript levels of *AtGA20ox2*, which plays a key role in GA biosynthesis. We observed a negative correlation between *CpSVP-Y* and *GA20ox2* transcript expression in the junction. *CpSVP* is upregulated in the junction tissue of *CpSVP-Y (1kb): CpSVP-Y* and *CpSVP-Y (2kb): CpSVP-Y*, while *GA20ox2* expression decreased an average of 4-fold for *CpSVP-Y (1kb): CpSVP-Y* and *CpSVP-Y (2kb): CpSVP-Y.* The *cis*-mutated lines did not vary in their *AtGA20ox2* expression pattern, suggesting that these *cis*-elements do not affect GA signaling and that the GA-related transcription factor does not interact directly with *CpSVP-Y* through these four *cis*-elements.

In *Arabidopsis*, *ASYMMETRIC LEAVES 2* (*AS2*) suppresses *BREVIPEDUNCLELUS (BP)* to control pedicel length [24]. *AS2* is downregulated in young pedicels in *CpSVP-Y (1kb):CpSVP-Y*, and the removal of P1 results in the upregulation in young pedicels. However, in *CpSVP-Y (2kb): CpSVP-Y*, removal of P1 results in the downregulation of *AS2* in young pedicels. This repressive mark could be due to *CpSVP (2kb*) possessing additional *cis*-regulatory elements that could be bound by transcription factor(s), which *CpSVP (1kb*) is missing. In tobacco, *SVP* interacts with *BP* to repress its expression, to regulate pedicel elongation [13]. *CpSVP-Y (2kb): CpSVP-Y* and *CpSVP-Y (1kb): CpSVP-Y* transgenic plants had higher *BP* expression than Col-0 when we introduced *CpSVP-Y.* Mutagenesis of the GA *cis*-elements decreased *BP* expression in the old pedicels compared to the non-mutated lines (Figure S6). As removal of the *cis*-elements subsequently increased *CpSVP-Y* expression and decreased *BP* expression to elongate pedicels, this suggests that this mechanism could be conserved and contribute to papaya peduncle elongation. However, further experiments are needed to confirm this mechanism.

In the 2kb *CpSVP* promoter region, *CpSVP-Y* and *CpSVP-Y^h^* are 99% similar. Hermaphroditic papaya is a product of human domestication 4000 years ago [2], so not enough time could have passed for drastic divergence from the male. In the 2kb *CpSVP* promoter region, males and hermaphroditic papaya differ in a single *cis*-element for a plastid response element, which acts as an enhancer in the Chlamydomonas *HSP70A* promoter and is induced by Mg-protoporphyrin IX (MgProto) and light [35]. MgProto is involved in the chloroplast retrograde signaling system. The floral repressor *FLOWERING LOCUS C (FLC)* is silenced by chloroplast retrograde signals to promote flowering under high light. Hermaphrodites have this *cis*-element intact and males have a 2 bp mismatch in this *cis*-element. In addition to other flowering genes, this could contribute to how hermaphrodites control the floral transition, as *CpSVP-Y^h^* is nonfunctional. With *CpSVP-Y’s* conserved role in flowering time, it could contribute to males flowering earlier than female and hermaphroditic plants. In addition to male papaya plants possessing an elongated peduncle, this helps to increase the spread of the Y chromosome in the population to maintain the high genetic diversity and increase fitness, and allows males to remain competitive against hermaphrodites.

The conclusion of this study is mainly based on the heterologous expression system of *Arabidopsis thaliana*. To directly verify the functions of these GA-related *cis*-elements in papaya, future work will utilize genetic transformation techniques to construct different deletion-line mutant plants in the male (XY) and hermaphroditic (XY^h^) backgrounds of papaya, and observe their direct effects on flower stalk length and flowering time. In addition, the direct determination of endogenous GA levels in flower stalks by LC-MS/MS, combined with transcriptome analysis to systematically identify the downstream gene networks regulated by these *cis*-elements, will help reveal the complete signal transduction pathways. Further in-depth understanding of the regulatory mechanism of this gene, through molecular design breeding to regulate plant structure and optimize reproductive allocation to increase yield and adaptability, provides a new theoretical basis for papaya breeding work.

## 4. Materials and Methods

### 4.1. Plant Material

*Arabidopsis thaliana* ecotype Col-0 was used in the study. All plants were grown in a chamber at 23 °C (16 h light) and 18 °C (8 h dark) with 50% humidity.

### 4.2. Flowering Time Measurements

For flowering time comparison, the number of rosette leaves was counted when the bolt of plants reached 2 cm tall. For pedicel length comparison, pedicel length was measured from seven independent T3 lines for each genotype. For each independent line, 8 plants were grown, and the top and bottom six pedicels of each plant were measured.

### 4.3. Plasmid Construction

The coding sequences of *CpSVP-Y* and *CpSVP-Y^h^* were blasted to papaya male and hermaphrodite pseudomolecules to determine the promoter region, and 2kb and 1kb sequences upstream of the start codon (ATG) of *CpSVP-Y* and *CpSVP-Y^h^* were extracted. *Cis*-regulatory elements were identified using PLACE [16] and PlantCARE [17]. These promoter sequences were amplified by PCR using sequence-specific primers (CpSVP-Y/Y^h^ (1kb) and CpSVP-Y/Y^h^ (2kb), Table S1) and subcloned into the vector pMDC162-GUS [36]. The coding sequences of *CpSVP-Y* and *CpSVP-Y^h^* were PCR amplified (CpSVP-Y and CpSVP-Y^h^ primers, Table S1), and subcloned into vector pMDC162 containing the promoter region of interest. For the site-directed mutagenesis, plasmids containing the promoter were used as a template to amplify with inverse primers to remove the *cis*-element of interest (Table S1). Insert sequences of each construct were confirmed by Sanger sequencing.

### 4.4. Plant Transformation and Analysis of Transgenic Lines

Constructs were transformed into *Agrobacterium tumefaciens* strain GV3101. *Arabidopsis* plants were transformed using the floral dip method [37]. At least 12 independent, positive T1 lines were obtained for each construct. Seeds were sterilized and sown on ½ strength MS plates with hygromycin and cefotaxime, stratified at 4 °C for 2 days before placing them into the growth chamber. Once the seedlings reached the four-leaf stage, they were transferred to soil. At least 12 T1 plants were grown for each construct. T2 seeds were screened for single-locus transgene insertion based on 3:1 segregation on selective media and were genotyped using gene-specific primers with Kapa3G Plant PCR Kit (Cat no. KK7252), according to the manufacturer’s protocol.

### 4.5. Quantitative RT-PCR (qPCR)

Total RNA was isolated using the Trizol (Invitrogen, Waltham, MA, USA) method according to manufacturer’s protocol. RNA concentration was determined using Nanodrop 2000 (Thermo Fisher Scientific, Waltham, MA, USA), and integrity was assessed on 1% agarose gel. First-strand cDNA was synthesized with one microgram of total RNA using the Maxima cDNA kit with dsDNase (Cat no. K1672, Thermo Fisher Scientific) following the manufacturer’s suggested protocol. The qPCR reaction consisted of1.6 μL cDNA, 0.5 μM forward and reverse primers, 10 μL PowerUp SYBR Green Master Mix (Cat no. A25741, Applied Biosystems, Waltham, MA, USA), and ddH20 on a CFX96 Touch Instrument (BioRad, Hercules, CA, USA). qPCR primers used are listed in Table S2, as previously described or designed with Primer3 software [38,39]. The PCR program consisted of an initial denaturation at 50 °C for 2 min and 95 °C for 2 min, then 35 cycles of amplification at 95 °C for 15 sec and 60 °C for 1 min, followed by a dissociation curve. A minimum of three independent T3 lines (2 technical repeats each) were included for each experiment. Data were normalized to *ACTIN2* and *ELONGATION FACTOR 1-α* using the ΔΔ Ct method [40].

### 4.6. Histochemical and Quantitative GUS Activity Assay

Histochemical staining of *Arabidopsis* GUS activity was conducted as previously described [41]. Various tissues from T3 transformants (leaf, inflorescence, stem, and pedicels) were collected 44 days after planting, and incubated in GUS staining solution (0.1 M NaPO_4_ at pH 7.0, 10 mM EDTA, 0.1% Triton X-100, 1 mM K_3_FE(CN)_6_ [Thermo Scientific], 2 mM X-Gluc [Gold Biotechnology, St. Louis, MO, USA]) overnight. Samples were cleared with 70% ethanol. Images were taken using a Zeiss AxioZoom V16 microscope (Zeiss, Jena, Germany) or Canon PowerShot SX50 HS (Canon Inc., Tokyo, Japan) camera and analyzed with FIJI software (ImageJ version 1.53t, https://fiji.sc/, accessed on 15 December 2025).

For GUS activity quantification, tissue (junction, young, and old pedicels) was collected from 44-day-old seedlings, flash frozen, and homogenized in GUS extraction buffer (100 mM KPO_4_ at pH 7.8, 1 mM EDTA, 1% Triton X-100, 10% Glycerol) for protein extraction. Total protein was quantified with Pierce 660 nm Reagent (Cat no. 1861426, Thermo Scientific), and standardized with bovine serum albumin. A total of 10 μL of protein sample and 200 μL GUS extraction buffer supplemented with 2.5 mM MUG were incubated at 37 °C for 60 min. Then, 1 M of sodium bicarbonate was added to stop the reaction. GUS activity was measured fluorometrically and normalized as per microgram of protein added. Readings were taken at excitation 360 nm and emission 460 nm using BioTek Synergy HTX Plate Reader with Gen 5 software. Four biological replicates and three technical repeats were assayed for each genotype.

## Data Availability

Data from experiments are presented in the figures and supplementary tables and figures.

## Supplementary Materials

The following supporting information can be downloaded at https://www.mdpi.com/article/10.3390/plants15020209/s1. Table S1. Plasmid construction primers. Restriction enzyme site is underlined; Table S2. Primers used in quantitative RT-PCR study; Table S3. Flowering time of translational and mutagenized translational constructs; Figure S1. Analysis of cis-acting regulatory elements in the 2-kb promoter regions of *CpSVP-Y* and *CpSVP-Y^h^*; Figure S2. Peduncle length for *CpSVP(2kb):CpSVP* and mutagenized *CpSVP(2kb):CpSVP* lines. 12 peduncles from 16 individual plants were measured for each T3 line and 7 independent lines were used in this study. Values are represented as mean peduncle length (in cm) ± standard error. Results were compared to Col-0 and analyzed with Student’s *t*-test (* *p* < 0.05, ** *p* < 0.01); Figure S3. Peduncle length for *CpSVP(1kb):CpSVP* and P1-removed *CpSVP(1kb):CpSVP* lines. peduncle length for complementation study. 12 peduncles from 16 individual plants were measured for each T3 line and 7 independent lines were used in this study. Values are represented as mean peduncle length (in cm) ± standard error. Results were compared to Col-0 and analyzed with Student’s *t*-test (** *p* < 0.01); Figure S4. Relative expression of *GA20ox2* transcript levels in translational and mutagenized translational lines. The qPCR expression level of each construct was normalized to *ACTIN* and *EIF1* expression in wild type Col-0 plants. Values were obtained from three biological replicates and two technical replicates; Figure S5. Relative expression of GUS transcript levels in native promoter and mutagenized lines. The qPCR expression level of each construct was normalized to *ACTIN* and *EIF1* expression in wild type Col-0 plants. Values were obtained from three biological replicates and two technical replicates; Figure S6. Relative expression of *AS2* and *BP* transcript levels innative promoter and mutagenized lines. The qPCR expression level of each construct was normalized to *ACTIN* and *EIF1* expression in wild type Col-0 plants. Values were obtained from three biological replicates and two technical replicates.
