# Supplementary material for: Identification of a GA-Related Cis-Element Regulating Male Peduncle Elongation in Papaya
Source: Plants (Basel). 2026 Jan 9;15(2):209. doi: 10.3390/plants15020209 (PMC12845159; doi:10.3390/plants15020209)
Supplement: Supplementary file 1 [file plants-15-00209-s001.zip › plants-4016105-supplementary.pdf]

Supplementary Table S1. Plasmid construction primers. Restriction enzyme site is underlined.

Name	Sequence (5' to 3')	Purpose
CpSVP-Y-F	GTACGGCGCGCCATGGCGAGGGAGAAGATAC	cloning, genotyping
CpSVP-Y-R	GCTAGGCGCGCCTTACCCAAGCTTTAGAGAG	cloning, genotyping
CpSVP-Y^h-F	GCTAGGCGCGCCATGAAGGAAATACTTGAAAG	cloning, genotyping
CpSVP-Y^h-R	GCTAGGCGCGCCTTACCCAAGCTTTAGAGAG	cloning, genotyping
CpSVP-Y/Y^h(1kb)-F	GTACTTAATTAAAAGTCTCAAACAATGATGCTGTCTG	cloning
CpSVP-Y/Y^h(1kb)-R	GCTAGGCGCGCCTCCTCTCTTCTCTTCTTCTCTTTCC	cloning
CpSVP-Y/Y^h(2kb)-F	CCCTTAATTAAAAGTCTCAAACAATGATGCTGTCT	cloning
CpSVP-Y/Y^h(2kb)-R	GCTAGGCGCGCCTCCTCTCTTCTCTTCTTCTCTTTCC	cloning
Hyg-F	CAAGCTCTGATAGAGTTGGTCAAGA	genotyping
Hyg-R	ATACACTACATGGCGTGATTTCATA	genotyping
1KB P461_F	CACTGTCTAGATATATTACTCATTGTAAC	Mutagenesis
1KB P461_R	GTTACAATGAGTAATATATCTAGACAGTG	Mutagenesis
1KB P895_F	GAGCTCAGAAAACATGATACTGACTCTTGATTTTG	Mutagenesis
1KB P895_R	CAAAATCAAGAGTCAGTATCATGTTTTCTGAGCTC	Mutagenesis
2KB G272_F	CTCTCATTAGCAGAGGCTGGAATATG	Mutagenesis
2KB G272_R	CATATTCCAGCCTCTGCTAATGAGAG	Mutagenesis
2KB PG272_F	CTCTCATTAGCAGCTGGAATATGAG	Mutagenesis
2KB PG272_R	CTCATATTCCAGCTGCTAATGAGAG	Mutagenesis

Supplementary Table S2. Primers used in quantitative RT-PCR study.

Name	Sequence (5' to 3')
AS2-F [39]	TGTTTGGTGGCTTGGATGTTCC
AS2-R [39]	AACAGTACGGCGACCATCATCT
BP-F [39]	GATCGGGAACTCAAGAACCA
BP-F [39]]	CATCCATCACCATGAACTGC
EF1-F [23]	ATGCCCCAGGACATCGTGATTTCAT
EF1-R [23]	TTGGCGGCACCCTTAGCTGGATCA
AtGA20ox2-F [22]	ACCGAGACTATTTCCGAGGATT
ATGA20ox2-R [22]	TGTTTGGCATGGAGGATAATG
AtActin2-F	CGCCATCCAAGCTGTTCTC
AtActin2-R	TCACGTCCAGCAAGGTCAAG
GUS-F	AACCGTTCTACTTTACTGGCTTTGG
GUS-R	GCATCTCTTCAGCGTAAGGGTAAT

Primers as described in Husbands et al. 2015 [38], Yamaguchi *et al.* 2007 [23] , and Rieu *et al.* 2008 [22]

Supplementary Table S3. Flowering time of translational and mutagenized translational constructs.

Genotype	Number of Rosette Leaves
WT (Col-0)	11.8 ± 0.16
CpSVP-Y^h(1kb):CpSVP-Y^h	11.9 ± 0.64
CpSVP-Y(1kb):CpSVP-Y	13.3 ± 0.29 **
CpSVP-Y(1kb):CpSVP-Y (P1)	11.8 ± 0.33
CpSVP-Y(2kb):CpSVP-Y	13.3 ± 0.25 **
CpSVP-Y(2kb):CpSVP-Y (P1)	12.3 ± 0.48
CpSVP-Y(2kb):CpSVP-Y (P2)	13.7 ± 0.18 **
CpSVP-Y(2kb):CpSVP-Y (P3)	12.6 ± 0.20 **
CpSVP-Y(2kb):CpSVP-Y (P4)	12.6 ± 0.21 **
pMDC162	12.5 ± 0.51

Eight individual plants from three independent lines were measured for each genotype. Values are means ± standard error. Asteriks indicate significant differences when compared to the WT. (**p<0.01, Student's t-test)

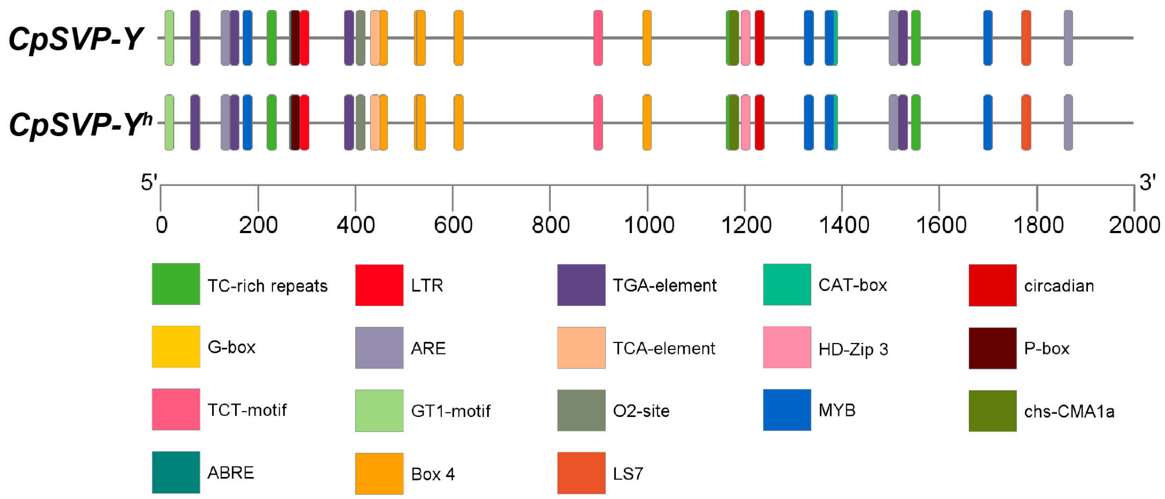

Supplementary Figure S1. Analysis of cis-acting regulatory elements in the 2-kb promoter regions of *CpSVP-Y* and *CpSVP-Y*^{h}.

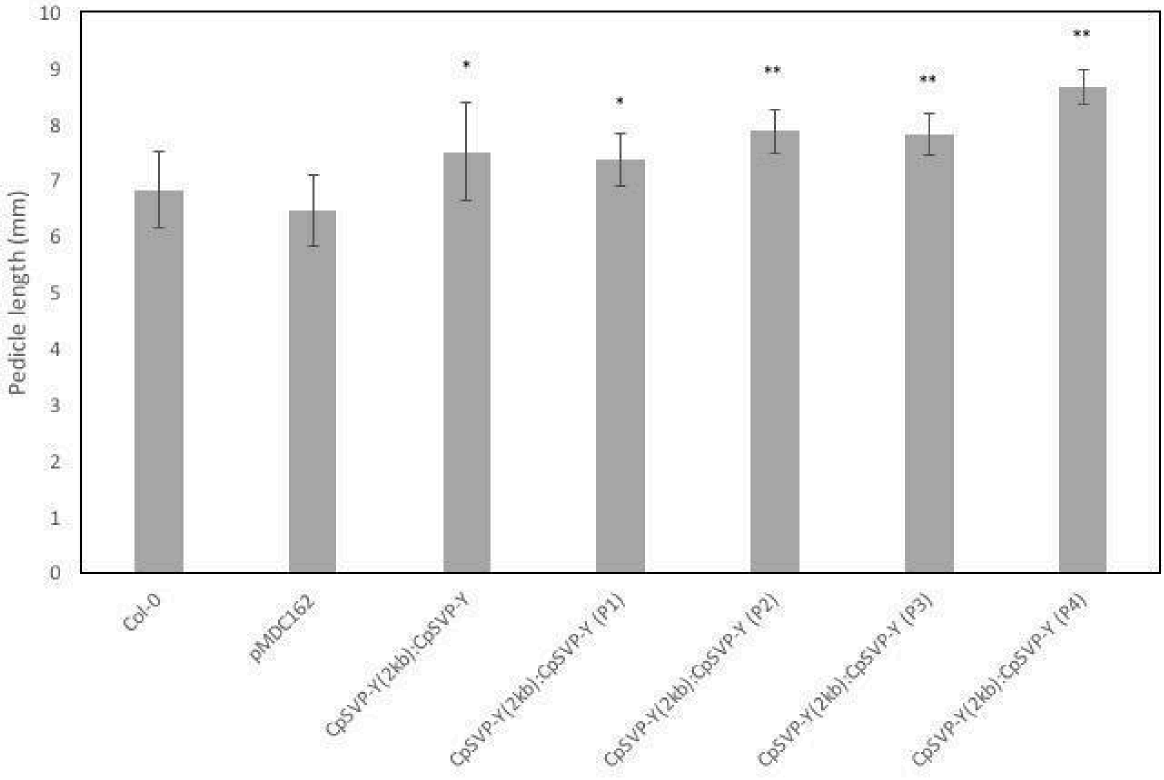

Supplementary Figure S2. Peduncle length for *CpSVP(2kb):CpSVP* and mutagenized *CpSVP(2kb):CpSVP* lines. 12 peduncles from 16 individual plants were measured for each T3 line and 7 independent lines were used in this study. Values are represented as mean peduncle length (in cm) ± standard error. Results were compared to Col-0 and analyzed with Student's t-test (**p*< 0.05, ***p*< 0.01).

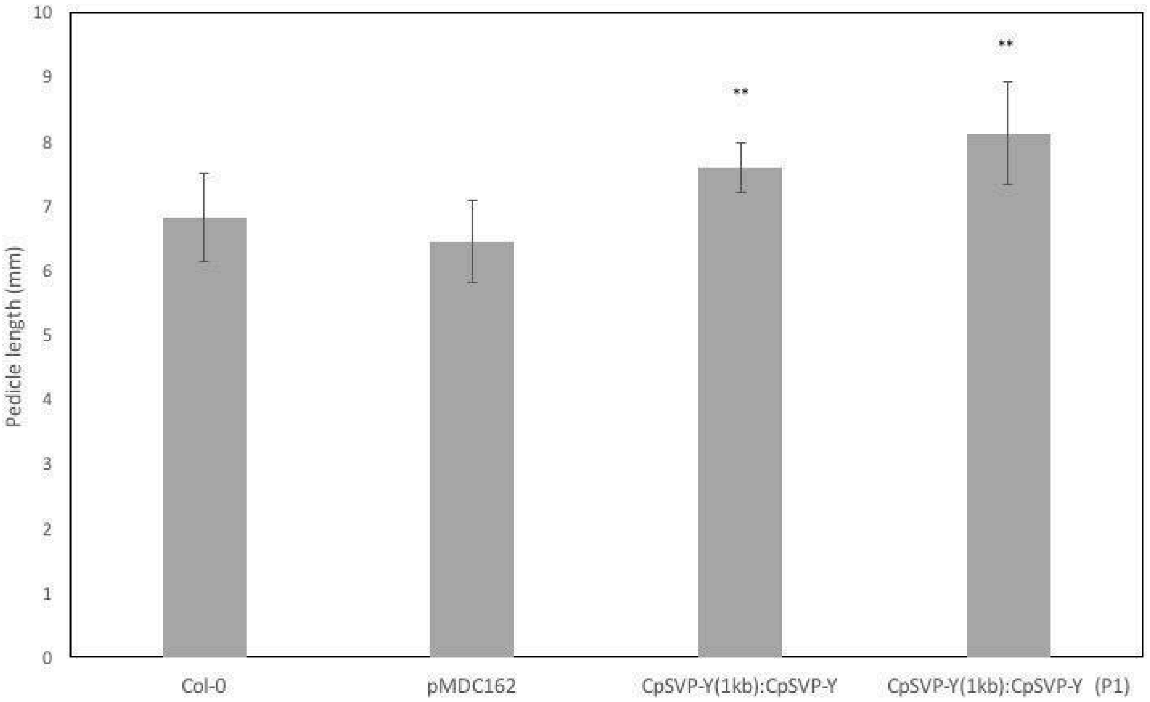

Supplementary Figure S3. Peduncle length for *CpSVP(1kb):CpSVP* and P1-removed *CpSVP(1kb):CpSVP* lines. peduncle length for complementation study. 12 peduncles from 16 individual plants were measured for each T3 line and 7 independent lines were used in this study. Values are represented as mean peduncle length (in cm) ± standard error. Results were compared to Col-0 and analyzed with Student's t-test (***p*< 0.01).

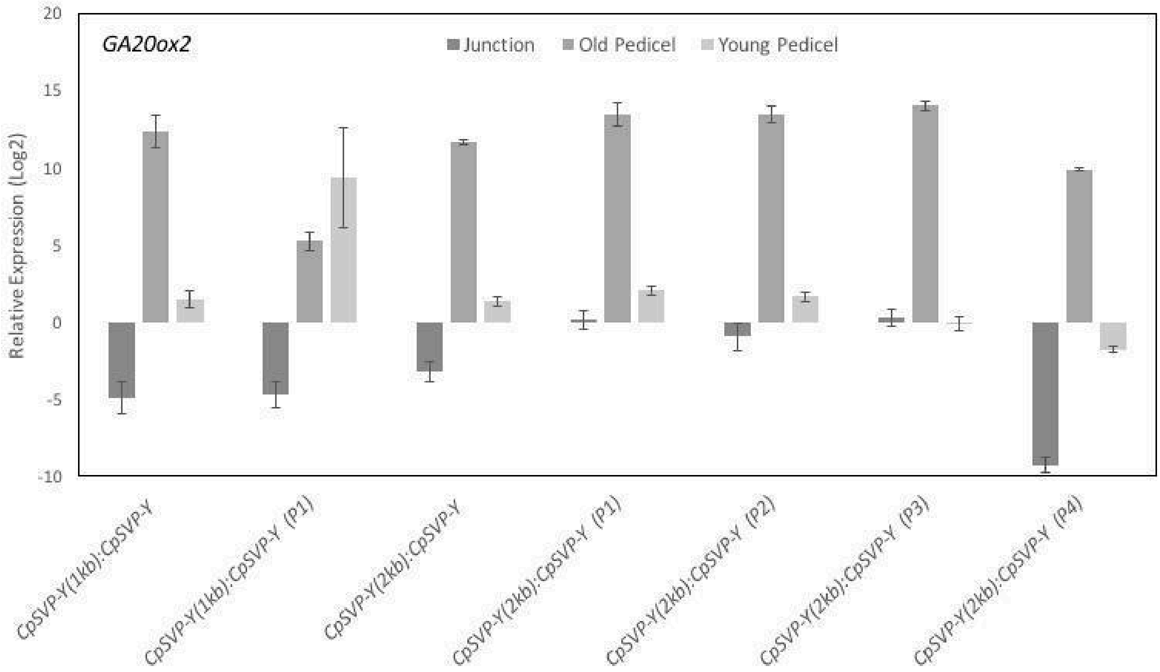

Supplementary Figure S4. Relative expression of *GA20ox2* transcript levels in translational and mutagenized translational lines. The qPCR expression level of each construct was normalized to *ACTIN* and *EIF1* expression in wild type Col-0 plants. Values were obtained from three biological replicates and two technical replicates.

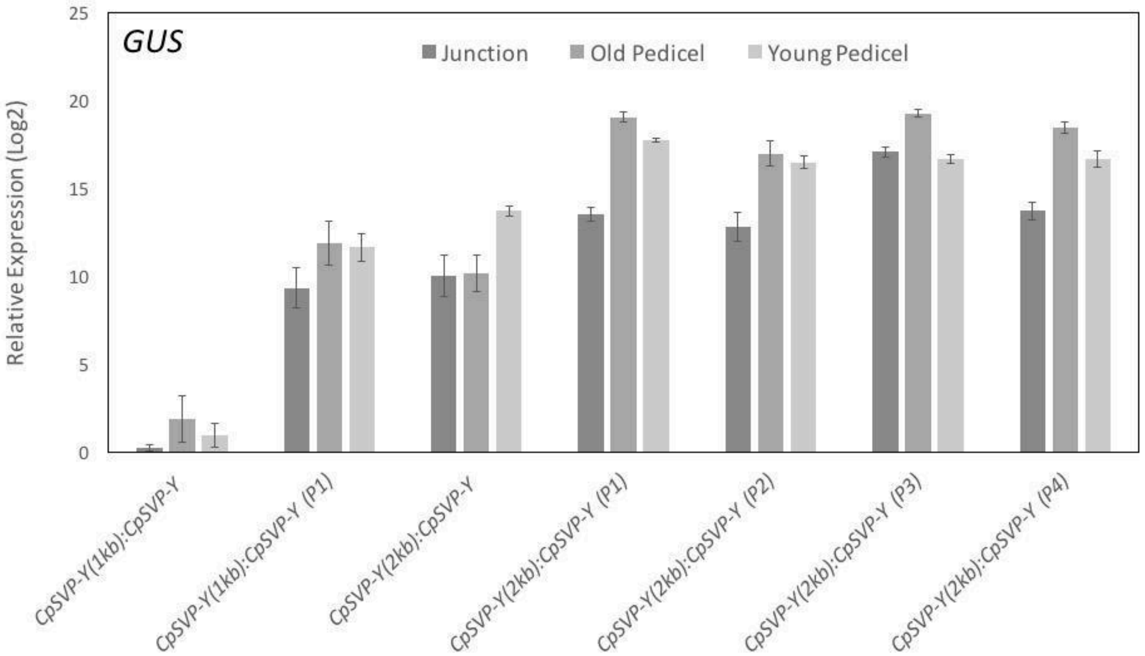

Supplementary Figure S5. Relative expression of GUS transcript levels in native promoter and mutagenized lines. The qPCR expression level of each construct was normalized to *ACTIN* and *EIF1* expression in wild type Col-0 plants. Values were obtained from three biological replicates and two technical replicates.

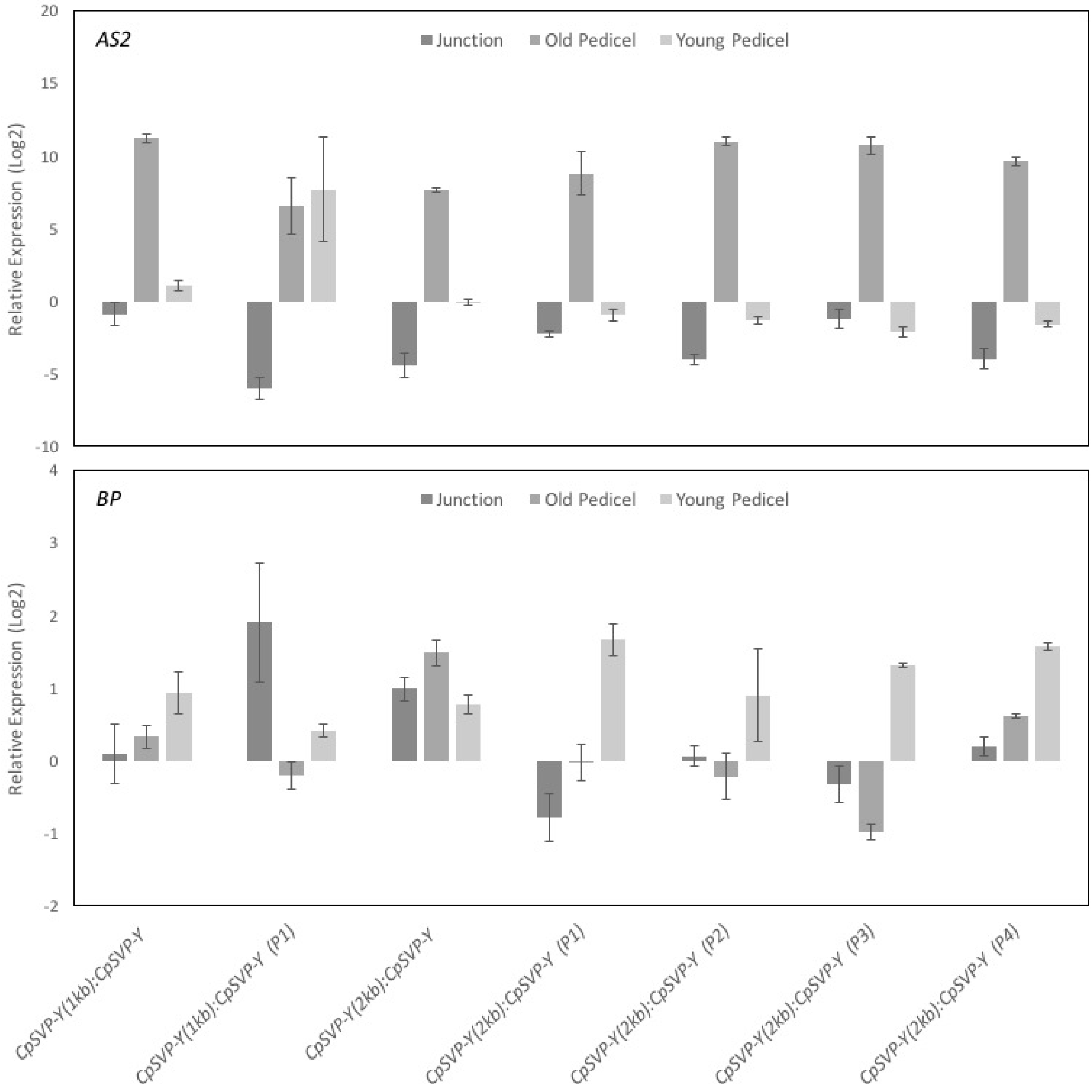

Supplementary Figure S6. Relative expression of *AS2* and *BP* transcript levels innative promoter and mutagenized lines. The qPCR expression level of each construct was normalized to *ACTIN* and *EIF1* expression in wild type Col-0 plants. Values were obtained from three biological replicates and two technical replicates.
